# Long-range magnetic interactions and proximity effects in an amorphous exchange-spring magnet

**DOI:** 10.1038/ncomms11931

**Published:** 2016-06-13

**Authors:** F. Magnus, M. E. Brooks-Bartlett, R. Moubah, R. A. Procter, G. Andersson, T. P. A. Hase, S. T. Banks, B. Hjörvarsson

**Affiliations:** 1 Department of Physics and Astronomy, Uppsala University, Box 516, Uppsala 751 20, Sweden; 2 Science Institute, University of Iceland, Dunhaga 3, Reykjavik IS-107, Iceland; 3 Department of Chemistry, University College London, 20 Gordon Street, London WC1H 0AJ, UK; 4 LPMMAT, Université Hassan II de Casablanca, Faculté des Sciences Ain Chock, Maârif B.P. 5366, Morocco; 5 Department of Physics, University of Warwick, Coventry CV4 7AL, UK

## Abstract

Low-dimensional magnetic heterostructures are a key element of spintronics, where magnetic interactions between different materials often define the functionality of devices. Although some interlayer exchange coupling mechanisms are by now well established, the possibility of direct exchange coupling via proximity-induced magnetization through non-magnetic layers is typically ignored due to the presumed short range of such proximity effects. Here we show that magnetic order can be induced throughout a 40-nm-thick amorphous paramagnetic layer through proximity to ferromagnets, mediating both exchange-spring magnet behaviour and exchange bias. Furthermore, Monte Carlo simulations show that nearest-neighbour magnetic interactions fall short in describing the observed effects and long-range magnetic interactions are needed to capture the extent of the induced magnetization. The results highlight the importance of considering the range of interactions in low-dimensional heterostructures and how magnetic proximity effects can be used to obtain new functionality.

## Abstract (web summary)

In thin film devices, ferromagnetic layers may couple indirectly over short length scales via induced magnetization in otherwise non-magnetic spacers. Here, the authors demonstrate a long range exchange coupling across a paramagnetic layer.

## Graphical abstract

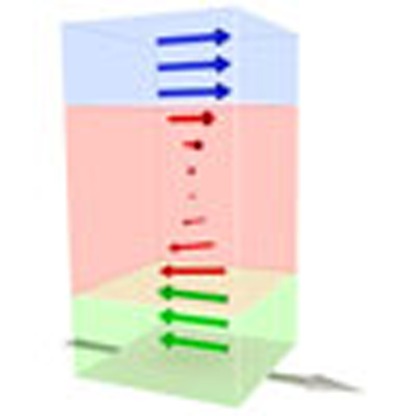

Magnetism is an intrinsically quantum mechanical phenomenon but classical approaches can nonetheless be very useful for describing certain magnetic properties^1^. Many features of ferromagnetic ordering such as the asymptotic changes in magnetization with temperature as well as the ordering temperature itself can be described well using atomistic models with only nearest-neighbour spin–spin interactions. However, as the spatial dimensions of the ferromagnet are reduced, the surfaces and interfaces, where the atomistic interactions are truncated, start to have a defining effect on the magnetic properties. Modelling such finite size effects has typically required the assumption of weakened or enhanced magnetic interactions at the boundaries, which approach the bulk state exponentially^2^. The need for such assumptions is removed if longer-range (beyond nearest-neighbour) magnetic interactions are allowed^3^,^4^,^5^,^6^,^7^. Although this approach is rarely used due to its computational complexity, it has been employed successfully to capture finite size effects on the magnetic ordering and describe the spatial variation of the magnetization in very thin, free-standing films^8^.

Extending these ideas to model magnetic heterostructures comprising multiple magnetic or non-magnetic layers can give a fresh insight into the interface phenomena, which are central to many current and emerging magnetic technologies. One of the most important, but often overlooked, interface phenomena in magnetic heterostructures is the magnetic proximity effect^9^. In general, this refers to the influence that an ordered magnetic state in one layer has on an adjacent layer^10^. When two magnetic materials are in direct contact, the influence of the proximity effects is mutual, leading to a variation in the coupling strength across the interface, which has been modelled previously using mean field theory^11^. This can for example give rise to changes in the ordering temperature of the two materials^12^ or even result in a single singularity in the susceptibility^11^. In another type of proximity effect between two ferromagnets with different directions of anisotropy, a mutual imprinting of domain structures has been observed^13^. Alternatively, at a ferromagnetic/non-magnetic interface a magnetization can be induced within the non-magnetic material. The extent of this proximity-induced magnetic region is typically quite short, of the order of only a few atomic distances, but in materials which are close to satisfying the Stoner criterion, such as Pd and Pt, it is known to extend up to a few nanometres^14^,^15^,^16^.

When two ferromagnetic layers are separated by a non-magnetic layer, proximity effects arise at both interfaces, which can give rise to long-range interlayer exchange coupling^16^, changes in ordering temperature^17^ and/or non-oscillatory alignment of the magnetic layers^15^,^18^. Here we provide experimental evidence that such a proximity effect can result in direct exchange coupling across a 40-nm-thick paramagnetic layer, which is more than an order of magnitude longer than previously demonstrated. The proximity-induced magnetization can give rise to both spring magnet behaviour and exchange bias, with the two having a different temperature dependence and extension. The extent of the proximity-induced magnetization and the richness of the resulting magnetic interlayer coupling effects are of importance for any magnetic metallic multilayered system. Furthermore, we show how such a proximity effect can be rationalized using an atomistic spin model with long-range magnetic interactions and thus move beyond the common assumption of only nearest-neighbour magnetic interactions.

## Results

### Magnetization measurements

**Figure 1:**
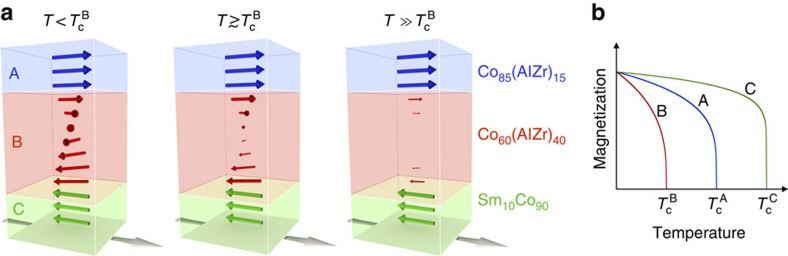
Design of the experiment. (**a**) A simplified schematic of the layer structure of the sample, showing the three different magnetic layers, A, B and C. Layer C has a large imprinted uniaxial anisotropy (parallel to the large grey arrow), whereas layer A has a small imprinted anisotropy (in the same direction) and layer B is isotropic. The magnetization profile during magnetization reversal, in different temperature regimes, is shown by the round coloured arrows, demonstrating the exchange-spring magnet behaviour and the magnetic proximity effect. The large grey arrow shows the direction of the applied magnetic field. (**b**) An illustration of the temperature dependence of the magnetization in the three layers, showing the three different ordering temperatures 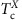 (*X*=A, B or C) of the layers.

Our experimental system is an amorphous exchange-spring magnet^19^, composed of a magnetic trilayer as illustrated in Fig. 1a. Such an amorphous heterostructure is ideal for examining magnetic interface effects, as amorphous materials form highly homogeneous and flat layers^20^, which are free of step edges and grain boundaries. A strong uniaxial anisotropy is imprinted^21^ in the bottom layer (C) and the ferromagnetic ordering temperature of the three layers 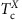 (*X*=A, B or C) is, through the choice of composition, as illustrated in Fig. 1b. The anticipated effect of the ferromagnetic proximity in the paramagnetic layer B can be seen in Fig. 1a. At low temperature 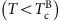 a normal spring-magnet behaviour is expected, whereas at high temperature 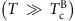 the top layer (A) is expected to act independently of the bottom layer (C). At intermediate temperatures 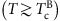 a ferromagnetic coupling may exist between layers A and C, arising from the proximity-induced ferromagnetism in layer B. The trilayer architecture and material choice are crucial, as they allow us to determine the range of any proximity-induced magnetization in layer B by determining the temperature dependence of the coupling between layers A and C for different thicknesses of layer B. However, as the ordering temperature of layer B and the coercivity of layer C can be tuned through composition, the temperature ranges and interaction strengths could be changed as desired.

**Figure 2:**
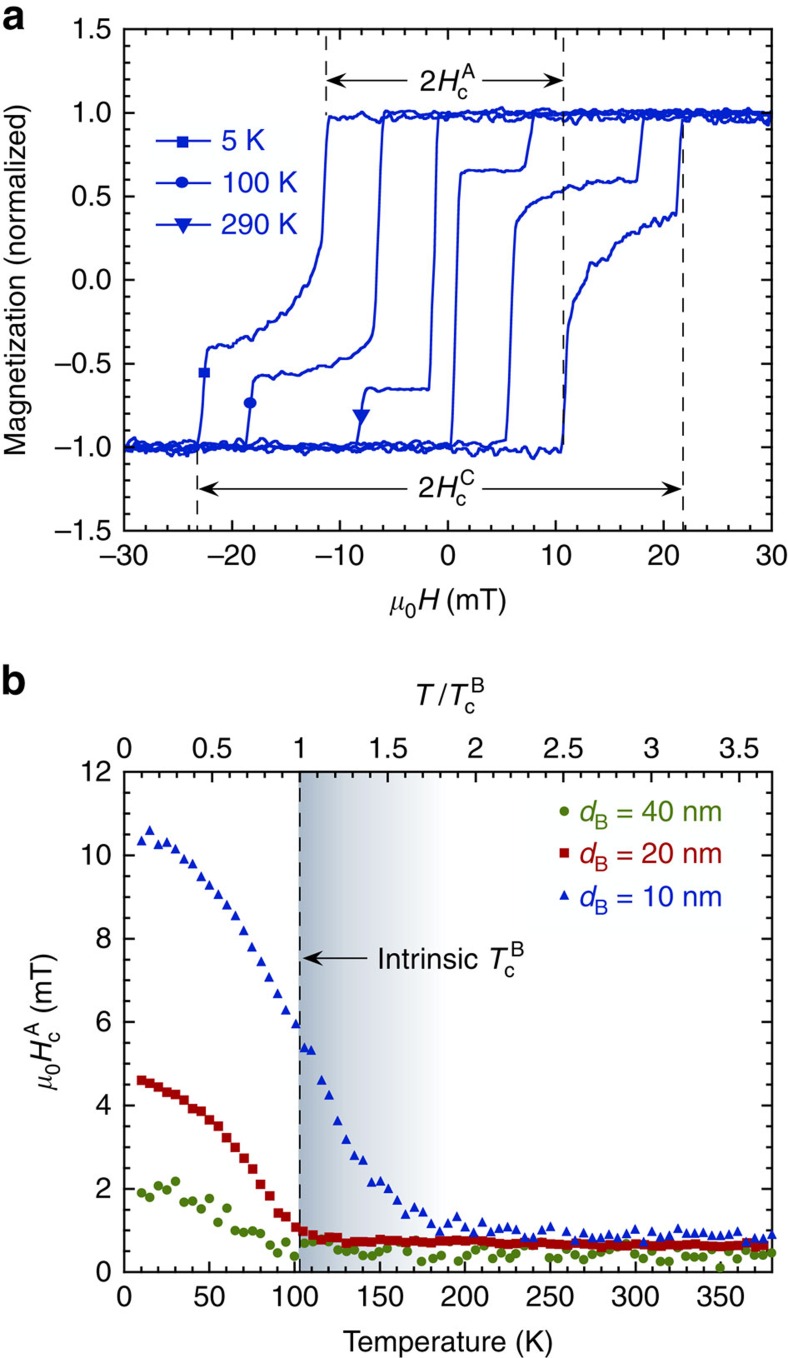
Spring-magnet behaviour and enhanced coercivity. (**a**) The magnetization along the easy axis for three different temperatures, showing the exchange coupling between the top and bottom layers. The middle layer thicknesses *d*_B_ is 10 nm. (**b**) The lower coercive field 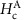 as a function of temperature (absolute and normalized by 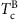), for three different middle layer thicknesses *d*_B_. The dashed vertical line indicates the intrinsic transition temperature of Co_60_AlZr_40_. A coupling between the top and bottom layers is seen in a region well above the intrinsic transition temperature of the middle layer when *d*_B_=10 nm (highlighted by the blue shaded area).

Representative easy axis magnetic hysteresis loops of a trilayer structure with a middle layer thickness *d*_B_=10 nm are shown in Fig. 2a, measured by magneto-optical Kerr effect (MOKE) magnetometry. Layer C (Sm_10_Co_90_) has a large imprinted in-plane uniaxial anisotropy resulting in a square magnetization loop with substantial coercivity along the easy magnetic axis^22^. Comparatively, layer A (Co_85_AlZr_15_) has a small in-plane anisotropy and layer B (Co_60_AlZr_40_) is isotropic, although the magnetization is constrained to lie in the plane of the films by the shape anisotropy. As the growth temperature is above the ordering temperature of layer B, no anisotropy is imposed during the growth of that layer. At room temperature (290 K), layer B should be paramagnetic (see Supplementary Fig. 1) and the A and C layers act independently and display switching fields 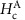 and 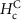, respectively. Consequently, the magnetic contributions of the two layers simply add, resulting in a two-step, square hysteresis curve as seen in Fig. 2(a). When the temperature is lowered below 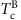 the magnetic coupling between adjacent layers causes the magnetization of the soft A and B layers to be pinned by the strongly anisotropic layer C. Therefore, the coercivity of layer A is strongly enhanced as compared with its value determined from an isolated film. A two-step switching is again observed where the lower switching field 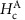 can be associated with the flipping of the top part of the trilayer (mostly layer A), whereas the high-field switch 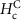 corresponds to the flipping of the magnetization of the bottom part of the trilayer (mostly layer C). In between the two switching fields, the characteristic exchange-spring magnet behaviour is observed with a gradual increase in the magnetization^19^. This gradual increase in the magnetization arises from the continuous in-plane rotation of the magnetization between the flipped top layer and the pinned bottom layer (analogous to a torsion spring), shown schematically in Fig. 1a.

For the B layer thicknesses *d*_B_=40 nm and *d*_B_=20 nm, the rise in coercivity occurs at the intrinsic transition temperature of layer B (103 K), as seen in Fig. 2b. Strikingly, when the thickness of the middle layer is reduced to *d*_B_=10 nm, the coupling between the top and bottom layers persists to above 150 K, which is >50% above the intrinsic ordering temperature of layer B (Co_60_AlZr_40_). The coupling can also be seen in the susceptibility when the field is applied parallel to the hard axis (see Supplementary Fig. 2). This apparent change in 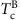 can not be attributed to finite size effects as the smallest thickness of *d*_B_=10 nm could only account for changes in *T*_c_ of 1–2% compared with the bulk^23^,^24^. Therefore, it is clear that the proximity of the ferromagnetic A and C layers to the intrinsically paramagnetic B layer induces some ferromagnetic ordering and an associated exchange stiffness within layer B, far above its intrinsic transition temperature. At 150 K, the proximity-induced ferromagnetic state extends at least 5 nm into the layer from both interfaces. With an interatomic distance of 0.15 nm, this corresponds to well above 30 atomic distances.

The two coercive fields 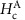 and 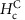 can be related to the energy barrier between the spiral and collinear magnetic states. The coercivity of layer C is at least an order of magnitude smaller than its saturation field along the hard axis (see ref. 22) and the reverse is true for the top layer (at least at low temperature). Therefore, the switching energy of both layers is associated with the domain wall forming within the trilayer. As the precise position of the domain wall and the magnetization profile within layer B is not known, we cannot calculate these energies precisely. However, using the coercive fields of layer A and layer C, and the associated magnetizations (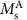 and 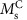), we can estimate the energy barrier using 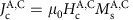. This gives an energy barrier of 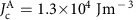 for the switching of the A layer and 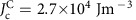 for the C layer at 5 K. These energies are highly temperature dependent as Fig. 2 shows.

**Figure 3:**
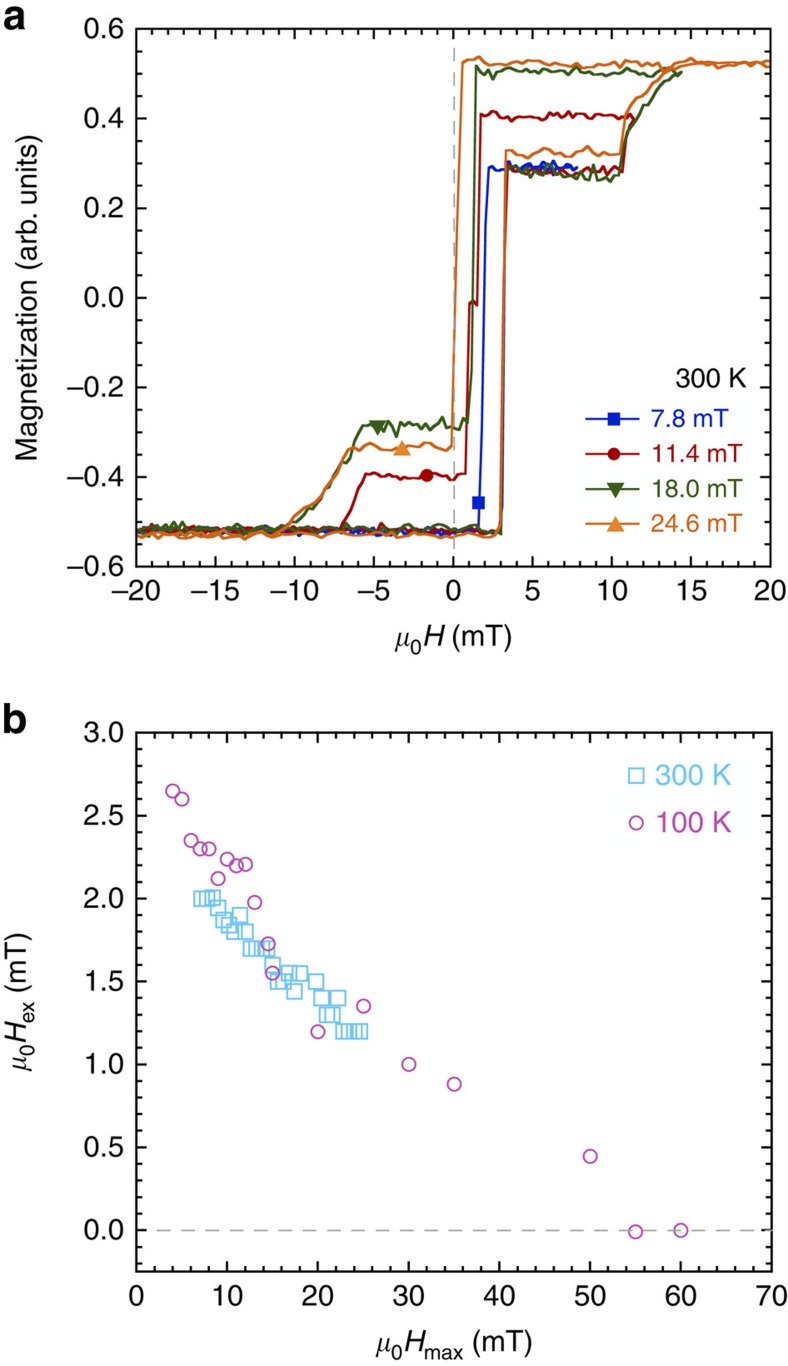
Exchange bias. (**a**) Room-temperature element-specific minor magnetization loops of Co, measured by X-ray resonant magnetic scattering, for successively higher maximum applied field in the positive direction *H*_max_. The sample is a trilayer with *d*_B_=40 nm. The loops are shifted towards positive field (as highlighted by the dashed line at zero field), showing the presence of exchange bias. (**b**) The exchange bias *H*_ex_ as a function of *H*_max_ for both 100 and 300 K. The exchange bias is the same at both temperatures and tends to zero (dashed line) as *H*_max_ increases.

Element-specific X-ray resonant magnetic scattering has been carried out to further examine the influence of proximity on the magnetic response in the thick layer limit (*d*_B_=40 nm). Measurements of the Co and Sm magnetization loops were performed at 100 K (at 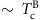) and 300 K. The results showed two-step switching of the Co and rounding at low temperature, in good agreement with the MOKE results. Minor loops were obtained by successively increasing the maximum positive field, as shown in Fig. 3a for the Co. There is no signature of exchange-spring behaviour or enhanced coercivity at high temperature, which is consistent with the MOKE data. However, the minor loops are clearly shifted to positive fields, demonstrating that layer A is in fact exchange biased by the high anisotropy layer C, even though there are no exchange-spring effects observed in hysteresis loops taken to complete saturation. We note that even the switching fields of layer C are shifted, implying that the Co in the bottom layer is also exchange biased. Comparing the Sm and Co magnetization loops reveals that the Co in the SmCo layer switches in smaller fields than the Sm, as described in detail elsewhere^25^. This decoupling of the Sm and Co sub-networks within the SmCo layer therefore results in an exchange bias from the Sm sub-network acting on the Co. The Co in the C layer then in turn results in an exchange bias in layer A.

The exchange field *H*_ex_ decreases as the maximum applied field increases as seen in Fig. 3b and is equal in size at both 100 and 300 K. This decrease in *H*_ex_ with applied field is a signature of the gradual switching of the Sm, which is weakly temperature dependent. Owing to this complex magnetization switching mechanism and the unknown magnetization profile within layer B, we can only estimate the coupling strength. Using the magnetization of layer A only 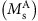 and the maximum obtained exchange bias field (from Fig. 3b) we find the coupling strength at 300 K to be approximately *J*_ex_=4 × 10^−5^ J m^−2^ using the relation 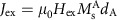. In any case, these data show that layer A is still coupled to layer C at 300 K, or three times the intrinsic ordering temperature of the 40-nm-thick B layer.

### Monte Carlo simulations

To examine the root cause of the observed proximity effects we have carried out Monte Carlo simulations of a model trilayer structure, resembling the sample shown in Fig. 1. A simple cubic model is used for simplicity and we adopt a terminology where a layer comprises a number (one or more) of identical monolayers. The simulations are based on a single crystal approach, which at first glance appears inconsistent with the experimental conditions. However, the modelling can be viewed as a rationalization of an arbitrary sample, where each atom represents a given volume fraction. Thus, the results can be scaled and generalized to capture the experimental findings, but are also applicable to other materials systems. A range of coupling schemes were used, from simple nearest neighbour up to eighth nearest neighbour, as described in the Methods section. In each case, the temperature was varied from well below to well above the ordering temperature of layer B.

**Figure 4:**
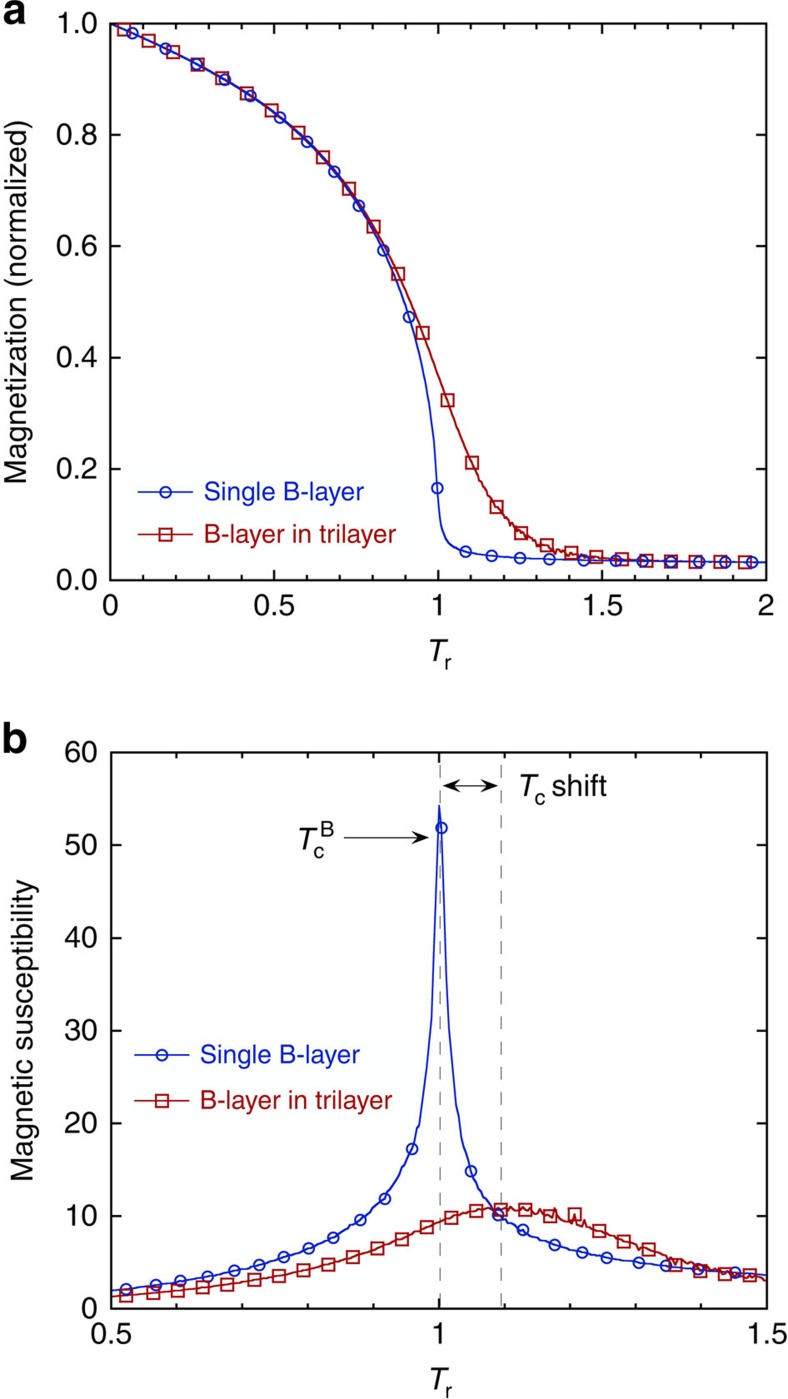
Simulations of *T*_c_ in a trilayer. (**a**) The magnetization versus temperature in the middle of a single B-layer and in the middle of a B-layer sandwiched between an A and C layer. The magnetization is calculated by including up to eighth nearest-neighbour interactions and the temperature is normalized by the intrinsic ordering temperature of the B layer found in the simulations 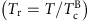. (**b**) The susceptibility of the middle atomic layer of a single B-layer and in the middle of a B-layer, sandwiched between an A and C layer. The shift in the ordering temperature of the sandwiched layer due to the proximity to the A and C layers can be seen clearly. Only a small subset of symbols is shown.

When using a nearest-neighbour interaction scheme, the induced magnetization in the paramagnetic layer was restricted to the near-interface region, which is clearly not consistent with the experimental results. Consequently, we will emphasize the influence of the range of interactions on the extent of the proximity effects. The temperature dependence of the magnetization and susceptibility of the eighth nearest-neighbour model is shown in Fig. 4. Two cases are considered: (i) a monolayer in the centre of a free-standing thin film of B (40 monolayers thick) and (ii) a monolayer in the centre of a B layer sandwiched by ferromagnetic A and C layers (10, 8 and 10 monolayers thick, respectively). Central monolayers are chosen as they are least affected by surface effects and for similar reasons, the properties of the monolayer in case (i) is considered to represent the bulk properties of the B layer. The temperature dependence of the magnetization for case (i) is as expected for the model as seen in Fig. 4a and the temperature dependence of the susceptibility (Fig. 4b) shows a sharp peak at the ordering temperature. The magnetization of the B-layer in the trilayer structure (case (ii)) shows a slower approach to the high-temperature behaviour, resembling the response of a ferromagnet in an external field (see Supplementary Fig. 1). The behaviour of the susceptibility suggests that the *T*_c_ of the middle monolayer of B is increased by ∼10% when the B-layer is sandwiched between layers A and C. More extensive finite size analysis would be required to determine this shift more precisely. The peak in the susceptibility is also significantly broadened, similar to the effect of applying an external magnetic field.

**Figure 5:**
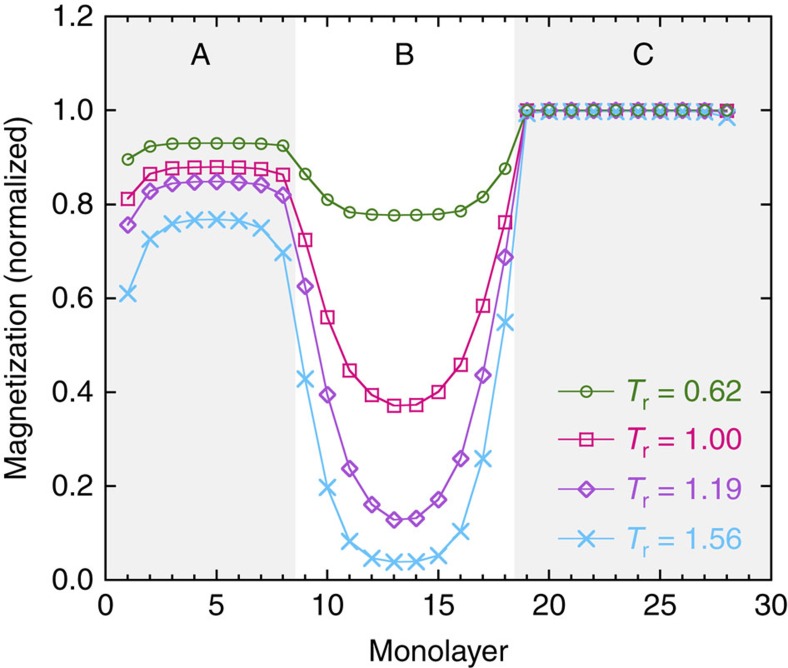
Magnetization profile in a trilayer. The simulated magnetization profile throughout the trilayer for a few temperatures above and below the 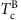 of a single B-layer. The temperature is normalized by the intrinsic ordering temperature of the B layer found in the simulations 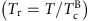. The magnetization decays into layer B away from the interfaces but a significant magnetization extends through the layer well above 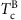.

Figure 5 shows the magnetization profile for the trilayer for a selection of temperatures. As the intrinsic ordering temperature of layer B (*T*_r_=1) is reached, a rapid reduction in the layer-resolved magnetization is seen, decaying from the interfaces into the centre of layer B. Despite this, layer B exhibits an observable magnetization at and somewhat above its intrinsic ordering temperature. Only for temperatures above *T*_r_=1.5 does the magnetization reach 0 in the centre of layer B. We also note that significant ferromagnetic proximity effects are present at the interfaces at temperatures that are as high as twice the ordering temperature of layer B.

**Figure 6:**
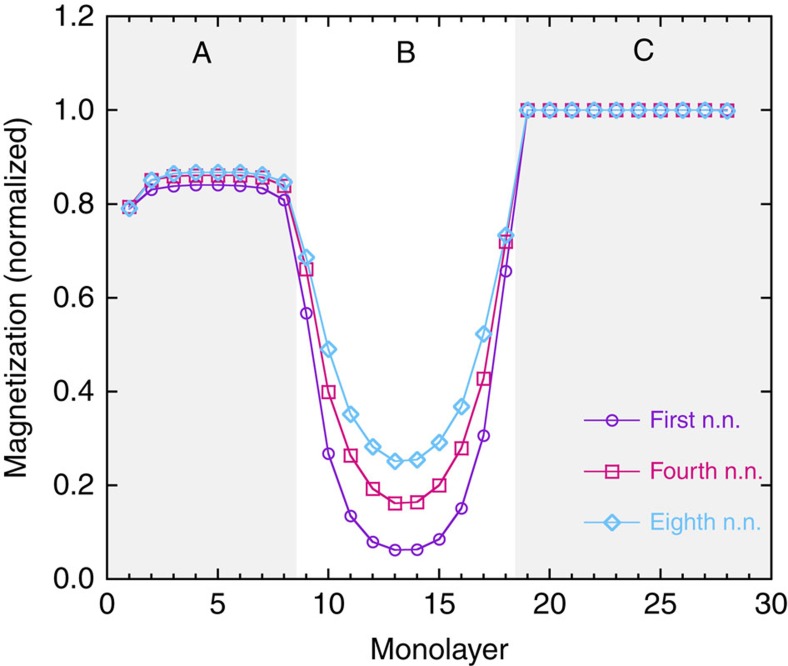
Range of interaction. The magnetization throughout the trilayer for different interaction ranges: first, fourth and eighth nearest neighbours (n.n.), at 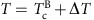, where 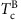 corresponds to the bulk ordering temperature for the respective range and Δ*T* is arbitarily chosen as 0.20. Monolayer 1 and 28 are the top and bottom surfaces of the sample, respectively. The background colour is a guide to the eye, showing the trilayer structure. A longer-range interaction is needed to capture the proximity-induced magnetization in the middle layer.

The extent of the proximity-induced magnetization and the ability to sustain this induction for 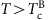 is greatly influenced by the range of the direct exchange interactions. This effect is illustrated in Fig. 6, which shows the magnetization profile in the trilayer structure for three different interaction schemes: nearest neighbour (corresponding to the first coordination sphere of each spin), up to fourth nearest neighbour (corresponding to the second coordination sphere) and up to eighth nearest neighbour (corresponding to the third coordination sphere). The profiles for each range are plotted at 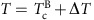, where 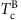 corresponds to the bulk ordering temperature for the respective range and Δ*T* is arbitarily chosen as 0.20. It is clear that a nearest-neighbour model does not capture the extent of the proximity-induced magnetism in the paramagnetic layer (B) that is determined experimentally when 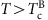. We also note that the proximity effect increases with increasing range of interaction in the simulations. From the regions near the interfaces, where there is little overlap between the proximity tails from either side of the B layer, we can estimate that the extent of the proximity region increases approximately linearly by a factor of 0.65 monolayers with the coordination sphere number. From this, one can estimate that the simulations would need to include up to the 45th coordination sphere, to obtain an extension of the induced magnetization of 30 atomic distances from the interfaces, but this is not feasible with the current computational methods.

## Discussion

The simulations show that an induced magnetization can be obtained well above the intrinsic ordering temperature of the B layer through proximity-induced magnetization. However, despite allowing interactions up to eighth nearest neighbours, our simulations underestimate the extent of the regions with induced magnetization. Increasing the range of interactions increases the extent of the proximity-induced magnetization, suggesting that even longer-range interactions are at play. However, another possible contribution to the observed range of the induced magnetization are atomic correlations within the amorphous layers. The disordered atomic structure can contain a modulation in the Co density, with interconnected regions of higher Co composition than the average. The presence of a modulation in the atomic specific density^26^,^27^,^28^ will result in regions of larger exchange coupling than the average coupling *J*_BB_ assumed within the B layer and are therefore more susceptible to the proximity of the adjacent ferromagnetic layers. Apparent structural disorder has indeed been shown to result in enhanced magnetic correlations above the ordering temperature in amorphous magnetic thin films with an associated high magnetic polarizability^29^,^30^. Therefore, it is possible that amorphous materials can exhibit larger proximity effects than their crystalline counterparts.

Independent of the root cause of the proximity effect, there are clearly two distinct coupling regimes, which have a different temperature dependence and extension. In the temperature regime 100<*T*≲150 K, there are ∼5-nm-thick regions in the B layer at its interfaces with an induced magnetization and an exchange stiffness, which exerts a torque on the A layer, enhancing its coercivity and resulting in spring-magnet behaviour. This can be considered as the proximity-induced ferromagnetic region. At greater distances from the interfaces and at temperatures up to at least room temperature, the B layer is still strongly polarized, although the spin stiffness is small or zero. This can be considered as the proximity-induced superparamagnetic region, where there is no torque on the A layer but the internal field results in an exchange bias effect. This state can be thought of as a magnetic liquid state in layer B. It is important to note that the temperature regions of these magnetic phases will be composition dependent and can thus be chosen for specific applications.

The results raise a number of fundamental questions about the range of magnetic interactions and the effect of structural disorder on magnetic properties. For example, from the results of the Monte Carlo simulations it appears that long-range interactions are the key to obtaining a substantial extension of a proximity-induced magnetization at interfaces. This has far reaching consequences concerning simulations of confined magnetic systems, not least the temperature dependence of the magnetization. Furthermore, the large extent of the proximity effect and its importance for exchange-spring behaviour and exchange bias imply that it needs to be considered in a range of structures showing interlayer exchange coupling. Finally, the two distinctly different regions of observed magnetic coupling, with different temperature dependence and extent, hint at the existence of a rich magnetic phase diagram for amorphous materials and an extensive scope for tailoring of their properties. This tuneability through temperature or composition can, for example, allow the increase of operating frequencies in microwave devices^31^, increase the performance of exchange-spring layer recording media^32^,^33^ or even add new functionality in areas such as magnetic sensors or logic, where a controllable interlayer coupling is desired. As a result, amorphous magnetic films may have an important role to play in future spintronic devices.

## Methods

### Sample growth and characterization

The samples were grown by dc magnetron sputtering in a sputtering chamber with a base pressure below 5 × 10^−10^ Torr. The sputtering gas was Ar of 99.9999% purity and the growth pressure was 2.0 mTorr. Si(100) substrates with the native oxide layer were used, 0.5 mm thick and with an area of 10 × 10 mm^2^. To remove surface impurities, the substrates were annealed in vacuum at 550 °C for 30 min before growth. First, a 2-nm-thick buffer layer of AlZr was deposited on the substrate from an Al_70_Zr_30_ alloy target of purity 99.9%. The buffer layer promotes the flat amorphous growth of the following layers. Subsequently, a 20-nm-thick Sm_10_Co_90_ alloy film was grown by co-sputtering from 2” elemental targets of Co (99.9% purity) and Sm (99.9% purity), after which a Co_60_(AlZr)_40_ layer in the thickness range 10–40 nm and a Co_85_(AlZr)_15_ layer of 15 nm were grown by co-sputtering from the Co and AlZr targets. Finally, a 3-nm-thick capping layer of AlZr was grown to protect the magnetic trilayer from oxidation. The simplified sample structure can be seen in Fig. 1a. All films were grown at room temperature, without any substrate cooling. The sample holder is equipped with two permanent magnets, which give a magnetic field of approximately *H*_im_=0.1 T parallel to the plane of the films. This magnetic field induces a uniaxial in-plane anisotropy in the layers, which are magnetic at room temperature. The sample holder design is described in detail in ref. 21. Structural characterization, attesting to the amorphicity of all layers and layer perfection, has been performed by grazing incidence X-ray diffraction, X-ray reflection and transmission electron microscopy (see ref. 22 for more details).

The magnetic characteristics of the samples were determined by magneto-optical Kerr effect (MOKE) measurements in the longitudinal geometry with *s* polarized light. The sample was rotated around the azimuthal angle *φ* (around the sample normal) and the full hysteresis loop recorded at 5° intervals, to determine the in-plane magnetic anisotropy. Full hysteresis loops were also recorded over the temperature range 5–380 K, for *φ*=0° and *φ*=90° with respect to the magnetic easy axis.

X-ray magnetic reflectivity was performed on the X13A beamline at the National Synchrotron Light Source^34^, using circular polarized X-rays tuned to the Co L_3_ edge. Keissig fringes with a period corresponding to the total trilayer thickness were observed, confirming that we are probing the entire trilayer thickness. Hysteresis loops were recorded by measuring the field dependence of the asymmetry ratio=(*I*^−^−*I*^+^)/(*I*^+^*I*^−^), with *I*^±^ the scattered intensity for X-rays of opposite helicity. In this geometry, the signal measured is element specific and sensitive to the in-plane ferromagnetic moment^35^. All loops were fitted to a modified Langevin function to quantify the coercivity and exchange bias.

### Model

The aim of the Monte Carlo simulations is to investigate the extent to which a simple, exchange-only, classical spin model can capture the essential physics of the experimental sample. Our model consists of a simple cubic lattice trilayer with periodic boundaries in the (*x*, *y*) plane and free boundaries in the *z* direction. The spin dimensionalities within each layer have been chosen as realistic representations of the magnetic moments in the layers. Using the labelling introduced in Fig. 1, layers A and B consist of only XY spins, constrained by the shape anisotropy to lie in the (*x*, *y*) plane of the lattice. Layer C contains Ising spins, constrained to point in only the ±*y* direction due to the strong uniaxial anisotropy.

The Hamiltonian governing our model is defined by the equation


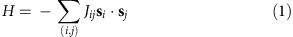


where the sum is over all pairs of spins with *i*≠*j* and the exchange couplings decay algebraically up to a hard cutoff at *r*=*r*_c_,


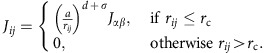


The spins, **s**, are three-dimensional vectors where one or more components may be fixed as zero to constrain the spin dimensionality (or type). In all cases the spins are of length 1. The system consists of *N*=28,672 spins, 1,024 per 32 × 32 layer with 8 layers of A spins and 10 layers each of B and C spins. The exchange parameter *J*_*αβ*_ is a member of the set (*J*_AA_, *J*_BB_, *J*_CC_, *J*_AB_ and *J*_BC_) according to the layers to which spins *i* and *j* belong.

The reduced energy scale of the exchange couplings is normalized to *J*_CC_=1.0. Other inter- and intra-layer couplings were then chosen as *J*_AA_=1.0, *J*_BB_=0.40 and *J*_AB_=*J*_BC_=0.90. These choices reflect the relative magnetic content (mole fraction of cobalt) of the layers, together with the fact that experimental results indicate that coupling within the B layer is significantly weaker than both of the inter-species couplings (Fig. 2).

The algebraic decay of the exchange couplings varies as 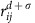 after Fisher *et al.*^36^, where *d* is the dimension of the system and *σ* is positive (and generally small). Although our slab is finite in the *z* direction, we assume that it is sufficiently large to take *d*=3. We initially simulated a range of *σ* between 0.1 and 2.0. Qualitatively, the results showed no variation and here we report the case *σ*=0.5. A fast decay (*σ*>2) in the interaction^37^ implies an approach to the nearest-neighbour model (the short-range limit). Our choice avoids this limiting behaviour, although allowing the further neighbour interactions to decay sufficiently quickly such that finite size effects are minimized (relative to the critical system at *σ*=0)^37^.

In our simulations we used a single spin-flip Metropolis algorithm with 10^4^ Monte Carlo steps per spin for equilibration and 10^5^ Monte Carlo steps per spin for observation at each temperature. We believe the spatial extent in the *xy* plane, 32 × 32 spins, is a reasonable compromise between accuracy and computational expense. Systems of this size allowed for accurate modelling of the critical phase of the two-dimensional *XY* model^38^ and it is known that finite size effects are smaller in three-dimensional systems^39^.

### Data availability

The data that support the findings of this study are available from the corresponding author upon request.

## Additional information

**How to cite this article:** Magnus, F. *et al.* Long-range magnetic interactions and proximity effects in an amorphous exchange-spring magnet. *Nat. Commun.* 7:11931 doi: 10.1038/ncomms11931 (2016).

## Supplementary Material

Supplementary Information Supplementary Figures 1-2

## References

[b1] Feynman R., Leighton R. & Sands M. The Feynman Lectures on Physics vol. 2, Addison-Wesley Publishing Company (1965).

[b2] Binder K. & Hohenberg P. C. Surface effects on magnetic phase transitions. Phys. Rev. B 9, 2194–2214 (1974).

[b3] Domb C. & Dalton N. W. Crystal statistics with long-range forces: I. The equivalent neighbour model. Proc. Phys. Soc. 89, 859–871 (2002).

[b4] Zhang R. & Willis R. Thickness-dependent Curie temperatures of ultrathin magnetic films: effect of the range of spin-spin interactions. Phys. Rev. Lett. 86, 2665–2668 (2001). 11290006 10.1103/PhysRevLett.86.2665

[b5] Luijten E. & Blöte H. W. Monte Carlo method for spin models with long-range interactions. Int. J. Mod. Phys. C 06, 359–370 (1995).

[b6] Luijten E., Blöte H. W. J. & Binder K. Medium-range interactions and crossover to classical critical behavior. Phys. Rev. E 54, 4626–4636 (1996). 10.1103/physreve.54.4626 9965639

[b7] Luijten E. & Blöte H. W. J. Classical critical behavior of spin models with long-range interactions. Phys. Rev. B 56, 8945–8958 (1997).

[b8] Taroni A. & Hjörvarsson B. Influence of the range of interactions in thin magnetic structures. Eur. Phys. J. B 77, 367–371 (2010).

[b9] Manna P. K. & Yusuf S. M. Two interface effects: exchange bias and magnetic proximity. Phys. Rep. 535, 61–99 (2014).

[b10] White R. M. & Friedman D. J. Theory of the magnetic proximity effect. J. Magn. Magn. Mater. 49, 117–123 (1985).

[b11] Wang R. W. & Mills D. L. Onset of long-range order in superlattices: mean-field theory. Phys. Rev. B 46, 11681–11687 (1992). 10.1103/physrevb.46.11681 10003057

[b12] Van der Zaag P. J., Ijiri Y., Borchers J. A. & Feiner L. F. Difference between blocking and Néel temperatures in the exchange biased Fe_3_O_4_/CoO system. Phys. Rev. Lett. 84, 6102–6105 (2000). 10991134 10.1103/PhysRevLett.84.6102

[b13] Heldt G. *et al.* Topologically confined vortex oscillations in hybrid [Co/Pd]_8_-Permalloy structures. Appl. Phys. Lett. 104, 182401 (2014).

[b14] Cheng L. *et al.* Pd polarization and interfacial moments in Pd-Fe multilayers. Phys. Rev. B 69, 144403 (2004).

[b15] Lim W. L., Ebrahim-Zadeh N., Owens J. C., Hentschel H. G. E. & Urazhdin S. Temperature-dependent proximity magnetism in Pt. Appl. Phys. Lett. 102, 162404 (2013).

[b16] Gökemeijer N. J., Ambrose T. & Chien C. L. Long-range exchange bias across a spacer layer. Phys. Rev. Lett. 79, 4270–4273 (1997).

[b17] Bovensiepen U. *et al.* Two susceptibility maxima and element specific magnetizations in indirectly coupled ferromagnetic layers. Phys. Rev. Lett. 81, 2368–2371 (1998).

[b18] Gottwald M., Kan J. J., Lee K., Kang S. H. & Fullerton E. E. Paramagnetic Fe_*x*_Ta_1-*x*_ alloys for engineering of perpendicularly magnetized tunnel junctions. APL Mater. 1, 022102 (2013).

[b19] Fullerton E. E., Jiang J. S., Grimsditch M., Sowers C. H. & Bader S. D. Exchange-spring behavior in epitaxial hard/soft magnetic bilayers. Phys. Rev. B 58, 12193–12200 (1998).

[b20] Choi C.-M., Song J.-O. & Lee S.-R. Thermal stability of magnetic tunnel junctions with new amorphous ZrAl-alloy films as the under and capping layers. IEEE Trans. Magn. 41, 2667–2669 (2005).

[b21] Raanaei H. *et al.* Imprinting layer specific magnetic anisotropies in amorphous multilayers. J. Appl. Phys. 106, 023918 (2009).

[b22] Magnus F. *et al.* Tunable giant magnetic anisotropy in amorphous SmCo thin films. Appl. Phys. Lett. 102, 162402 (2013).

[b23] Huang F., Kief M., Mankey G. & Willis R. Magnetism in the few-monolayers limit: a surface magneto-optic Kerr-effect study of the magnetic behavior of ultrathin films of Co, Ni, and Co-Ni alloys on Cu(100) and Cu(111). Phys. Rev. B 49, 3962–3971 (1994). 10.1103/physrevb.49.3962 10011291

[b24] Xin X., Pálsson G. K., Wolff M. & Hjörvarsson B. Finite-size effects: hydrogen in Fe/V(001) superlattices. Phys. Rev. Lett. 113, 046103 (2014). 25105636 10.1103/PhysRevLett.113.046103

[b25] Procter R. A. *et al.* Magnetic leverage effects in amorphous SmCo/CoAlZr heterostructures. Appl. Phys. Lett. 107, 062403 (2015).

[b26] Sheng H. W., Luo W. K., Alamgir F. M., Bai J. M. & Ma E. Atomic packing and short-to-medium-range order in metallic glasses. Nature 439, 419–425 (2006). 16437105 10.1038/nature04421

[b27] Ma D., Stoica A. D. & Wang X. L. Power-law scaling and fractal nature of medium-range order in metallic glasses. Nat. Mater. 8, 30–34 (2008). 19060888 10.1038/nmat2340

[b28] Hirata A. *et al.* Direct observation of local atomic order in a metallic glass. Nat. Mater. 10, 28–33 (2010). 21102454 10.1038/nmat2897

[b29] Ahlberg M., Andersson G. & Hjörvarsson B. Two-dimensional XY-like amorphous Co_68_Fe_24_Zr_8_/Al_70_Zr_30_ multilayers. Phys. Rev. B 83, 224404 (2011).

[b30] Korelis P. T. *et al.* Finite-size effects in amorphous Fe_90_Zr_10_/Al_75_Zr_25_ multilayers. Phys. Rev. B 85, 214430 (2012).

[b31] Kuanr B. K. *et al.* Increasing operational frequency in microwave devices by using [SmCo/NiFe] multilayered structures. IEEE Trans. Magn. 43, 2648–2650 (2007).

[b32] Berger A. *et al.* Improved media performance in optimally coupled exchange spring layer media. Appl. Phys. Lett. 93, 122502 (2008).

[b33] Oezelt H. *et al.* Micromagnetic simulation of exchange coupled ferri-/ferromagnetic composite in bit patterned media. J. Appl. Phys. 117, 17E501 (2015).

[b34] Sánchez-Hanke C., Kao C. C. & Hulbert S. L. Fast-switching elliptically polarized soft x-ray beamline X13A at NSLS. Nucl. Instrum. Methods A 608, 351–359 (2009).

[b35] Hill J. P. & McMorrow D. F. Resonant exchange scattering: polarization dependence and correlation function. Acta Crystallogr. A52, 236–244 (1996).

[b36] Fisher M. E., Ma S.-K. & Nickel B. G. Critical exponents for long-range interactions. Phys. Rev. Lett. 29, 917–920 (1972).

[b37] Hayakawa H., Rácz Z. & Tsuzuki T. Ordering kinetics in systems with long-range interactions. Phys. Rev. E 47, 1499–1505 (1993). 10.1103/physreve.47.1499 9960168

[b38] Bramwell S. T. & Holdsworth P. C. W. Magnetization and universal sub-critical behaviour in two-dimensional XY magnets. J. Phys. Condens. Matter 5, L53–L59 (1993).

[b39] Binder K. Monte carlo study of thin magnetic Ising films. Thin Solid Films 20, 367–381 (1974).

