# Supplementary material for: Long-range magnetic interactions and proximity effects in an amorphous exchange-spring magnet
Source: Nat Commun. 2016 Jun 13;7:ncomms11931. doi: 10.1038/ncomms11931 (PMC4910021; doi:10.1038/ncomms11931)
Supplement: Supplementary Information — Supplementary Figures 1-2 [file ncomms11931-s1.pdf]

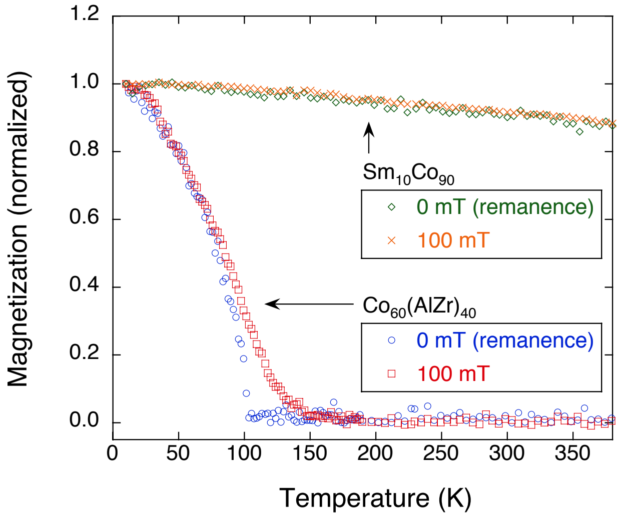

Supplementary Figure 1. **Magnetization of the trilayer constituents.** The 100 mT and remanent magnetization of single films of $Co_{60}(Al_{70}Zr_{30})_{40}$ and $Sm_{10}Co_{90}$ as a function of temperature. The $Sm_{10}Co_{90}$ magnetization changes only slightly in the temperature region studied. The $Co_{60}(Al_{70}Zr_{30})_{40}$ on the other hand has a ferromagnetic-paramagnetic phase transition at $T_c = 103 \pm 1$ K, as determined by fitting the remanent magnetization with the expression $M(T) = M_0\,(1 - T/T_c)^{\beta}$. The polarizability of the $Co_{60}(Al_{70}Zr_{30})_{40}$ is significant above its T_c as seen from the tail in the 100 mT magnetization extending up to approximately 150 K.

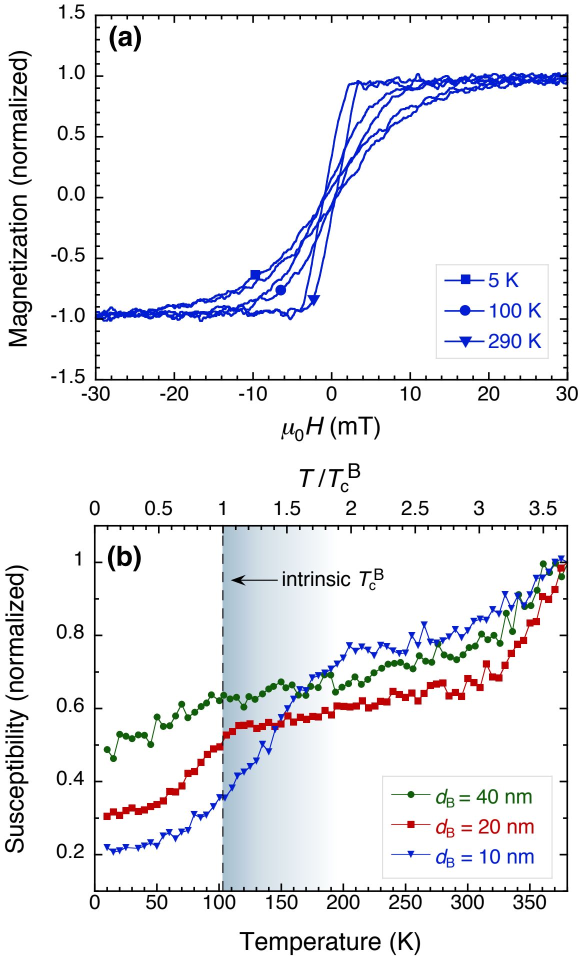

Supplementary Figure 2. **Hard axis magnetic response.** (a) The magnetization along the hard axis for three different temperatures, showing the exchange coupling between the top and bottom layers. The middle layer thickness d_B is 10 nm. For any given magnetic field below the saturation field, the longitudinal component of the magnetization will vary through the depth of the trilayer, depending on the spin stiffness. The spin stiffness will be largest for the C layer as it has the largest anisotropy. The field required to saturate the magnetization along the hard axis can be seen to decrease as the temperature is increased and the response becomes more linear, indicating that the variation in the degree of rotation throughout the trilayer is becoming smaller. (b) The zero field susceptibility (normalized to the susceptibility at the highest measurement temperature) as a function of temperature, for three different middle layer thicknesses d_B. The dashed vertical line indicates the intrinsic transition temperature of $Co_{60}AlZr_{40}$. The coupling between the top and bottom layers is reflected in the enhanced spin stiffness, which persists up to at least 180 K, well above the intrinsic transition temperature of the middle layer. This shows that in this region the spin stiffness of the A and B layers is still being enhanced by the coupling to the C layer, which can only be explained by a magnetic ordering being present within the B layer.
